# *In Silico* Identification of Plant miRNAs in Mammalian Breast Milk Exosomes – A Small Step Forward?

**DOI:** 10.1371/journal.pone.0099963

**Published:** 2014-06-16

**Authors:** Anna Lukasik, Piotr Zielenkiewicz

**Affiliations:** 1 Institute of Biochemistry and Biophysics, Polish Academy of Sciences, Warsaw, Poland; 2 Department of Plant Molecular Biology, Institute of Experimental Plant Biology and Biotechnology, University of Warsaw, Warsaw, Poland; CSIR Institute of Genomics and Integrative Biology, India

## Abstract

MicroRNAs (miRNAs) are a class of small RNA molecules that regulate gene expression by inhibiting the protein translation or targeting the mRNA cleavage. They play many important roles in living organism cells; however, the knowledge on miRNAs functions has become more extensive upon their identification in biological fluids and recent reports on plant-origin miRNAs abundance in human plasma and serum. Considering these findings, we performed a rigorous bioinformatics analysis of publicly available, raw data from high-throughput sequencing studies on miRNAs composition in human and porcine breast milk exosomes to identify the fraction of food-derived miRNAs. Several processing and filtering steps were applied to increase the accuracy, and to avoid false positives. Through aforementioned analysis, 35 and 17 miRNA species, belonging to 25 and 11 MIR families, were identified, respectively. In the human samples the highest abundance levels yielded the ath-miR166a, pab-miR951, ptc-miR472a and bdi-miR168, while in the porcine breast milk exosomes, the zma-miR168a, zma-miR156a and ath-miR166a have been identified in the largest amounts. The consensus prediction and annotation of potential human targets for select plant miRNAs suggest that the aforementioned molecules may interact with mRNAs coding several transcription factors, protein receptors, transporters and immune-related proteins, thus potentially influencing human organism. Taken together, the presented analysis shows proof of abundant plant miRNAs in mammal breast milk exosomes, pointing at the same time to the new possibilities arising from this discovery.

## Introduction

MicroRNAs (miRNAs) are a class of short (18–24 nt) regulatory RNAs that are widely evolutionary conserved among many species [1], [2]. These single-stranded, non-coding molecules mediate post-transcriptional gene regulation by promoting cleavage or inhibiting translation of the target mRNA [3], [4]. As a mature sequence form, miRNAs are generated in a multi-step process, which begins in nucleus from miRNA gene transcription into long primary transcript with many stem-loop units (pri-miRNA). The pri-miRNA is further processed into the hairpin precursor (pre-miRNA) and cleaved to generate the miRNA:miRNA* duplex with two nucleotide overhangs at the 3′ ends. In plants, these 2-nucleotide 3′-overhangs are then methylated by Hua Enhancer 1 (HEN1) methyltransferase [5], while in animals they remain unmethylated. In most cases, one of the duplex strands (*-strand) is degraded in the last stage of miRNA maturation process. Whereas, the second strand is loaded on the RISC (RNA-Induced Silencing Complex) multi-complex and binds to the specific mRNA transcript [6]. Throughout this hybridization, miRNAs negatively regulate expression of target genes, which control cell development, apoptosis, proliferation, differentiation and function in living organisms [7], [8]. Plant miRNAs not only play a role in organ development but also regulate nutrient homeostasis, environmental stress responses and phase changes [9], [10]. In humans, several reports have associated an expression profile of specific miRNAs with certain pathological stages, tumorigenesis or patient’s response to treatment. Thus, in medicine, miRNAs have become new diagnostic and prognostic biomarkers [11], [12], and have been incorporated in a few therapies for treating several human disorders [13], [14].

Growing interest in miRNAs and advancing experimental, and computational analytical approaches have contributed to a significant increase in information on miRNAs over the past few years. Using high-throughput sequencing methods, such as the Roche 454 Life Sciences System, Illumina Genome Analyzer and Applied Biosystems SOLiD system, along with many bioinformatics approaches, it is currently possible to identify large fraction of miRNAs, determine their expression level, predict precursor sequences, target genes and many other characteristics [15], [16]. The recent detection of miRNAs in body fluids (e.g., serum, urine, saliva, blood and milk) indicates that these molecules may play even greater role as gene expression regulators than initially anticipated [12], [17], [18]. The Gu *et al.* and Zhou *et al.* studies on miRNAs composition in porcine and human breast milk exosomes, respectively, demonstrated that resistant to harsh conditions, immune-related miRNAs are present and enriched in the examined membranous vesicles. Therefore, the authors suggest that breast milk exosomal miRNA molecules may be transferred to an infant’s body *via* the digestive tract and affect immune system development [19], [20]. Even more intriguing was a recent report on cross-kingdom regulation by plant miRNA, wherein the study by Zhang *et al.* provided evidence not only that exogenous, food-derived miRNAs are abundant in human serum but also that they can negatively regulate expression of specific genes in mammals. For example, MIR168a inhibits expression of the low-density lipoprotein receptor adapter protein 1 (LDLRAP1) in liver and thereby disrupts LDL plasma homeostasis [21]. The plant-origin miRNAs were also identified by the Wang’s *et al.* group, which showed that aforementioned molecules compose a significant sRNAs fraction in human plasma [22].

Considering the recent assumptions and evidences that endogenous and exogenous miRNAs might be sufficiently stable to pass through the gastrointestinal (GI) tract and enter circulation without losing functionality, we decided to do step forward, and determine whether plant miRNAs, especially those that were identified in serum, can be packed into mammalian breast milk exosomes. For this reason, we performed accurate bioinformatics analysis of the publicly available, raw data from small RNAs high-throughput sequencing studies on porcine and human breast milk exosomes. In the 12 datasets we successfully identified 17 and 35 miRNA species (from 11 and 25 MIR families), respectively. Additionally, to determine whether theses plant miRNAs may influence humans organism we predicted and annotated the target mRNAs that may potentially interact with the select miRNAs molecules.

## Results

### Small RNA Tags Analysis and Identification of Plant miRNAs in Breast Milk Exosomes

The raw data collected from 8 porcine and 4 human small RNA libraries included over 179.90 and 86.37 million reads, respectively. After removing the low-quality tags and contaminants as well as reads clustering, the analyzed datasets from the *H. sapiens* and *S. scrofa* included 1057,293/1,228,454/683,354/1,388,526 and 1,192,136/1,347,110/496,284/663,436/284,128/862,361/347,690/1,077,666 unique sequences, respectively.

**Figure 1:**
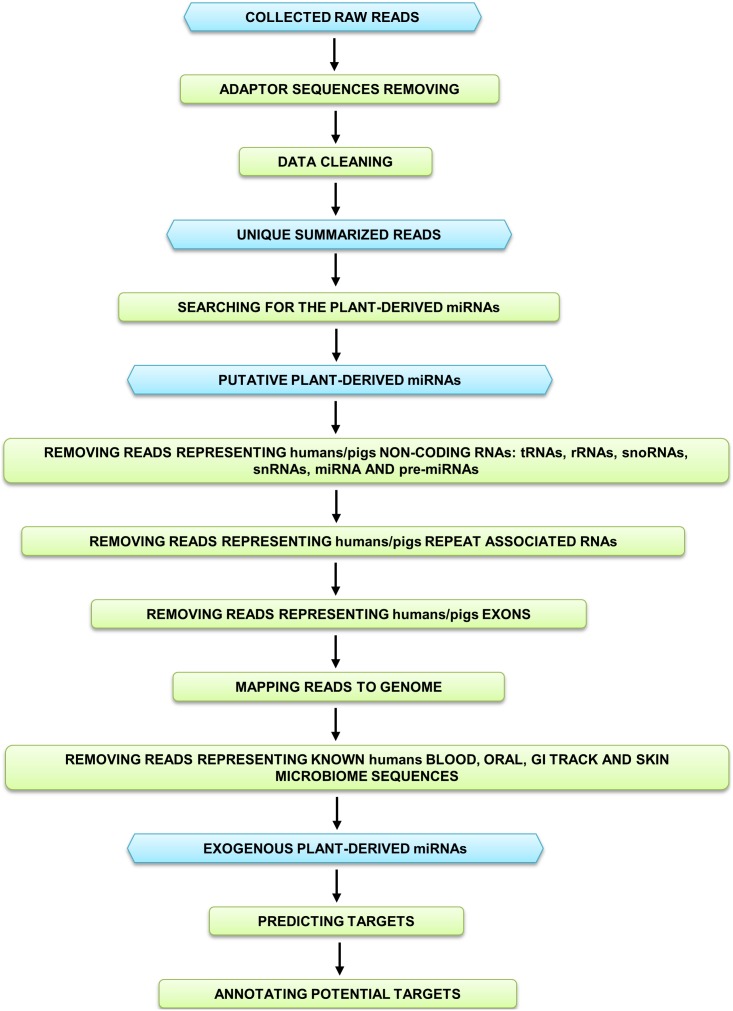
Workflow of human and pig breast milk exosomes sequencing data analysis. The reads collected from the 4 *H. sapiens* and 8 *S. scrofa* data sets were, each individually, cleaned and matched to known plant miRNAs to select all putative food-derived molecules. The matched tags were further subjected to few filtering steps, which resulted in elimination of all human and pig ncRNAs, repeat-associated RNAs, exon fragments and sequences successfully mapped to reference genomes, respectively. The remained reads were additionally examined to find and discard tags that with high probability represent specific microbiome sequences. As a second part of the analysis, the human targets prediction and annotation were carried out for select plant miRNAs. The aforementioned steps are detail described in the Materials and Methods section. Blue hexagons represent the data used and generated in the following processing/filtering steps (green rectangles) of the analysis.

**Figure 2:**
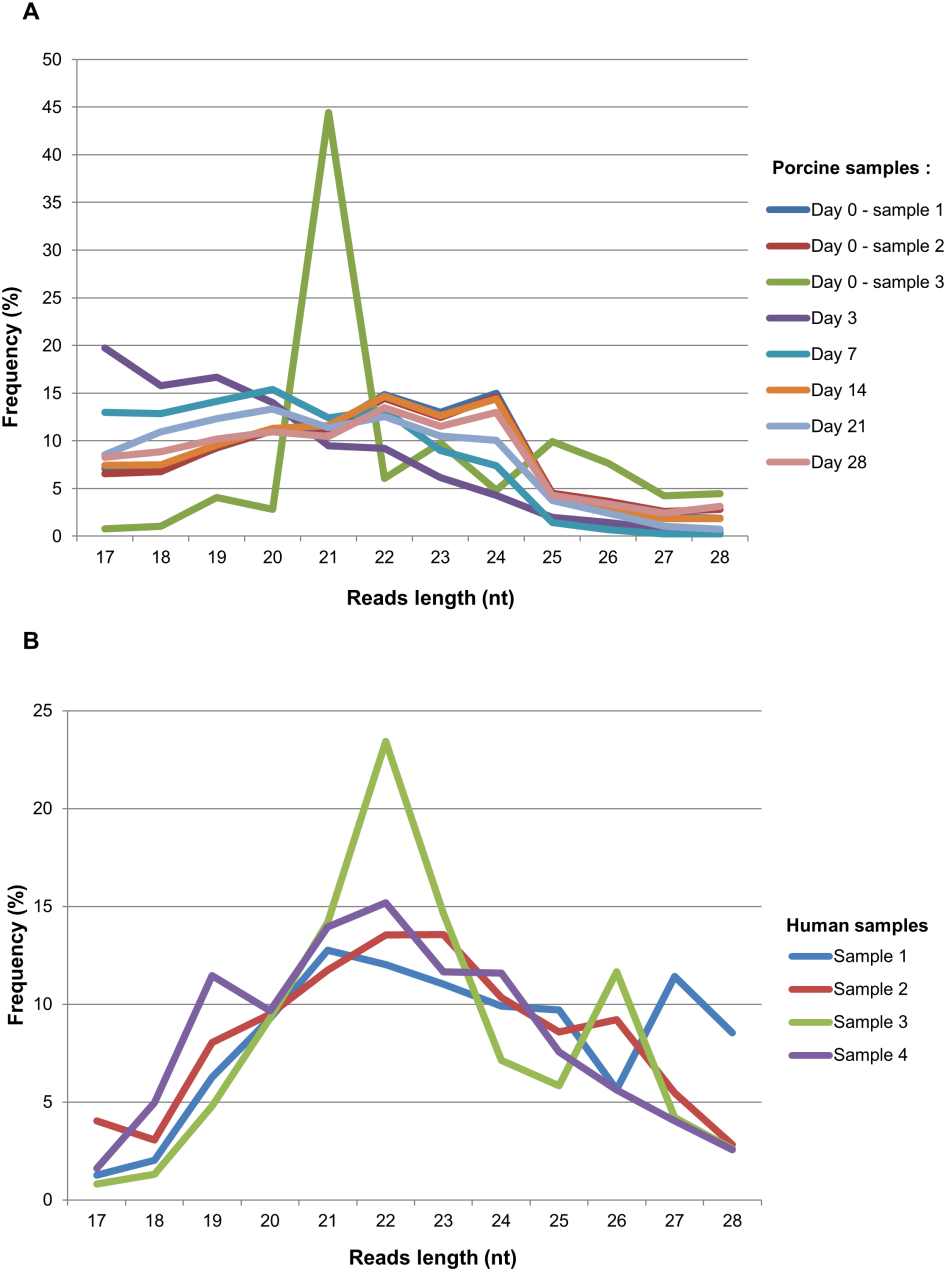
The lengths distribution examination of the exogenous-origin sRNA sequences. The summary of the sequence length distribution generated from (A) the *S. scrofa* and (B) *H. sapiens* tags, respectively, which remained after all processing and verification steps of the preformed bioinformatics analysis. Most of the generated reads were 21–24 nucleotides long.

The most important step in this study (the full workflow shown in Figure 1) was the identification of sequences with significant homology to plant miRNAs. Herein, the BlastN search against 10,597 miRNAs (from 127 plant species) and rigorous filtration of the obtained results were performed. In 8 *S. scrofa* and 4 *H. sapiens* samples, the initial number of potential plant miRNAs (with unique sequences) was 149/101/140/23/15/70/98/115 and 4,291/11,664/139/214, respectively. To discard the sequences with human and pig origins, the selected reads were verified in two steps. In the first, 26,846 *H. sapiens* and 3,834 *S. scrofa* ncRNA sequences were downloaded and supplemented with 155,394 and 64,365 mRNA sequences as well as the 1,350 and 769 repeat-associated RNA sequences, respectively. The putative plant miRNAs tags were then matched to this generated RNA datasets and significantly similar sequences were eliminated. For a second verification step, the reduced collections of potential exogenous miRNAs sequences from *S. scrofa* and *H. sapiens* were mapped to the pig and human genomes, respectively. In this procedure the common RNA editing modifications were taken into account. As a result of the aforementioned processes, several reads were discarded which yielded 120/86/49/0/0/56/60/69 and 751/2,435/0/5 total tags, respectively. In the final step of the described analysis, specific human microbiome sequences search did not yield any reads match. Therefore, the remaining tags collections were assumed to be plant-origin miRNAs, from which only molecules represented by five or more reads were considered. The length distribution examined for sequences classified as not originating from the *H. sapiens*, *S. scrofa* and human microbiome shown that most of these reads (from both species datasets) had 21 and 22 nucleotides (Fig. 2A,B).

From the bioinformatics analysis herein, 17 plant miRNA species that belong to 11 MIR families were identified in six porcine breast milk exosomes samples. The abundance levels of identified miRNAs were rather low, as well as the variations in plant miRNAs profiles across six samples. The matrix of calculated pairwise Pearson’s correlations is presented in Table 1 (average r = 0.230). Among the identified miRNA families, the MIR167, MIR319 and MIR444 composed the most members. The miRNA species with the highest abundance level (mean value from six samples) were as follows: zma-miR168a, zma-miR156a, ath-miR166a, ath-miR319b and ptc-miR319d.

**Table 1:** Pearson’s Correlation among Five Porcine Breast Milk Exosomes Samples.^a^

	Day 0 (Sample 1)	Day 0 (Sample 2)	Day 0 (Sample 3)	Day 14	Day 21	Day 28
**Day 0 (Sample 1)**		0.205 (0.336)	0.126 (0.575)	0.262 (0.238)	0.185 (0.398)	0.654 (0.0002)
**Day 0 (Sample 2)**			0.004 (0.984)	0.099 (0.651)	0.131 (0.590)	0.349 (0.080)
**Day 0 (Sample 3)**				0.214 (0.378)	0.616 (0.006)	0.013 (0.952)
**Day 14**					0.067 (0.769)	0.146 (0.494)
**Day 21**						0.391 (0.048)
**Day 28**						

a The correlation was calculated for each pair of samples, based on the counts of identified plant miRNAs. The P-values are given in brackets.

In contrast to the porcine data analysis, plant miRNA species were only identified in two out of four human breast milk small RNAs libraries; however, the Pearson’s correlation between these two samples indicated low changes in the identified plant miRNAs profiles (r = 0.675; P-value = 3.14e^−09^). As a result of the *H. sapiens* datasets examination, 35 miRNAs from 25 MIR families were detected, including one miRNA* - aly-miR157d*. The human and porcine plant miRNAs collections shows certain similarities. For example, the molecules with the highest abundance levels in the *H. sapiens* samples include ath-miR166a, pab-miR951, ptc-miR472a, bdi-miR168, aly-miR167d, osa-miR444b.2 and zma-miR156a, and the most numerous families were MIR166 and MIR167. The full lists of plant miRNA species/MIR families identified in porcine and human breast milk exosomes can be found in Tables S1–S8 in File S1 and Tables S9–S12 in File S2, respectively.

### Prediction and Annotation of Putative Human Target Genes for Plant miRNAs

MiRNAs functions in living organisms are associated with their binding to target mRNA, whereupon this mRNA is cleaved, or protein translation is inhibited. Thus, selecting and annotating miRNAs potential targets are the first steps in defining their roles in the cell. For initial insight into the probable influence of plant miRNAs on human organism and to determine whether they may regulate important biological processes, putative targets were predicted for the five select miRNAs with the highest abundance levels. The results generated by intersection of the miRanda, RNAhybrid and PITA method suggested that 1,282 unique human mRNAs are potential targets for the aforementioned plant miRNAs. These mRNA molecules include 369 mRNAs for ath-miR319b, 364 for ctr-miR167, 120 for ath-miR166a, 264 for zma-miR156a and 165 for osa-miR444b.2. The putative targets were further sorted, and the best 20–25 molecules with the highest alignment scores and lowest MFE of the structure were selected. The performed annotations using the Blast2GO and DAVID v6.7 software, with the additional KEGG pathway mapping, show that predicted targets included several mRNAs of proteins associated with the immune system function (e.g., ZEB1, IKAROS2, IL1-RAcPL and IL1RL1), molecules required for mediating hormone responses (e.g., NcoA-1 and MC4R), transcription factors and additional receptors relevant to an organism’s health (e.g., low-density lipoprotein receptor (LDLR), histamine receptor H2 (HRH2) and poliovirus receptor-related protein 4 (PVRL4)). Generally, the proposed human targets codes proteins involved in important biological processes, such as gene expression, steroid biosynthesis, transport, immune responses and starch, purine, and sphingolipid metabolism. The best predicted targets for the select plant miRNAs with their GO annotations, as well as the KEGG pathway mapping results are presented in Tables S13 and S14 in File S3.

## Discussion

Recent advances in experimental and computational analytical approaches have resulted in an explosion of information on miRNA molecules, which play many important roles in a wide range of organisms. Such methods have facilitated the identification of miRNAs profiles in human body fluids (e.g., saliva, blood, urine and milk), which served as biomarkers for detecting and monitoring various pathological conditions in certain circumstances [12], [17], [18]. Thus far, most studies have investigated host-origin small RNA molecules; therefore, discovering miRNAs from exogenous species in human serum was surprising and intriguing [21]. Zhang *et al.* identified food-derived, plant miRNAs, which were sufficiently stable to pass through human GI tract and enter the circulation. Moreover, *in vitro* and *in vivo* studies demonstrated that one of these molecules regulate expression of specific gene; thus, influencing certain molecular process in human organism [21]. The aforementioned study was not the only research that reported plant small RNAs in *H. sapiens* samples. Wang *et al.* also identified in human plasma a wide range of small RNAs from many different organisms, including food-derived miRNAs [22].

As for host-origin miRNAs, recent studies indicate that immune-related, endogenous miRNAs are enriched in breast milk exosomes. These molecules are resistant to relatively harsh conditions and thus, are assumed to influence infant immune system development [19], [20]. Considering aforementioned reports, the following question arises: can food-derived miRNAs pass through GI tract, enter circulation and pack into breast milk exosomes? To answer this question, we performed a rigorous bioinformatics analysis using publicly available, high-throughput sRNA sequencing data from mammalian breast milk exosomes. We processed 12 datasets (4 from *H. sapiens* and 8 from *S. scrofa*) and stringently verified the obtained results to avoid sequencing, and searching errors (Fig. 1). Herein, we eliminated the low-quality and endogenous-origin reads, as well as sequences highly likely to be part of the human microbiomes genomes. The tags that remained after these processing steps had length distributions typical for mature plant miRNA sequences; the most abundant reads had 21 and 22 nucleotides (Fig. 2A,B). This can be an additional indication that the aforementioned tags represent small, exogenous-origin RNAs [23]–[25].

We identified 17 plant miRNAs from 11 MIR families in the porcine breast milk exosomes. In turn, in human breast milk exosomes, we detected 35 exogenous miRNAs belonging to 25 plant MIR families (Tables S1–S8 in File S1 and Tables S9–S12 in File S2). The plant miRNAs profile compositions in breast milk exosomes from both species are consistent with Zhang’s *et al.* study on food-derived miRNAs in human serum [21]. MiRNAs identified at high levels in blood, such as MIR166a, MIR168a, MIR167d and MIR156a, were also highly abundant in mammalian breast milk exosomes. This observation suggests that plant miRNAs, which are sufficiently stable in serum, may be further transferred to milk exosomes. Moreover, the aforementioned miRNAs are evolutionarily conserved in diverse plant species (including those being an integral part of our daily diet) and typically appear at high levels in their various organs [23]–[27]. Thus, we can assume that even if a large fraction of the food-derived miRNAs was degraded along their pathway to mammal serum, the quantity of identified miRNAs was still sufficient to access the mammary glands and pack into the milk exosomes.

The accuracy of the carefully performed bioinformatics analysis reduced the initial potential plant miRNAs data from 149/101/140/23/15/70/98/115 and 4,291/11,664/139/214 to 120/86/49/0/0/56/60/69 and 751/2,435/0/5 total tags in porcine and human breast milk exosomes, respectively. The rather low abundance of these exogenous-origin molecules can be explained by the fact that data analyzed herein were derived from studies whose main goal was to identify mammalian miRNAs. The protocols and solutions employed in samples preparation, as well as sequencing procedure from aforementioned studies, met the known standards, and are commonly/successfully used in this type of experiments [28]–[32]. What is more important, they were constructed so as to enhance precision and efficiency in identifying animal miRNAs. It has been reported that the 2′-O-methyl modification of plant miRNAs 3′ ends can decrease the RNA ligase efficiency to ligate the adaptor oligonucleotides [33]. Therefore, sub-optimal parameters may reduce the number of plant miRNAs in the sequencing data. The results presented by Zhang *et al.* raised a debate among scientists; critics suggest that plant miRNAs in animal small RNA samples arise from cross-contamination, sequencing error and bias [34], [35]. However, it is unlikely that all plant miRNAs identified in animal datasets generated by different techniques and research groups may originate during sequencing or from contamination. In our study, the plant miRNAs have been identified in most, but not all, human and porcine breast milk exosomes samples; while, the correlation calculated between subsets, where these plant molecules were present, was moderate and low, respectively (Table 1). This highlights the high variation in plant miRNAs profiles from breast milk exosomes across different healthy individuals and may be an additional argument supporting the concept of food-derived plant miRNAs abundance rather than samples cross-contamination. Clearly, further analyses and experiments are necessary to resolve this issue and answer a more important question – whether the food-derived miRNA molecules influence human organism?

Endogenous miRNAs play many important roles in living organism cells, however exogenous-origin miRNAs may also regulate expression of specific genes and influence relevant biological processes [21]. Therefore, the second part of our study considers potential target prediction for five identified plant miRNAs with the highest abundance level - ath-miR319b, ctr-miR167, ath-miR166a, zma-miR156a and osa-miR444b.2. The targets were proposed by three independent methods (miRanda, PITA and RNAhybrid), which use different features for the miRNA target predictions. The obtained results were intersected, namely, the target was considered only if it was selected by all three algorithms. This kind of combining methods is a common practice to increase the precision/accuracy of predictions [36]–[39]. The proposed targets were further sorted using the highest alignment scores and lowest MFE of the structures; the top 20–25 hits were selected. Among the aforementioned, predicted molecules several mRNAs coding immune-related proteins were found, such as the transcription factor - zing finger E-box-binding homeobox 1 (ZEB1) [40]. The ZEB1 and ZEB2 mRNAs are known targets of human miR200 family members. The miR200 by inhibiting the ZEB1 and ZEB2 proteins production induce the mesenchymal-to-epithelial transition in cancer cell lines and reduce their aggressiveness [41]. Additional molecules that participate in organism’s immune responses include the receptor accessory protein of the IL-18 (IL-18RAcPL) and interleukin-1 receptor-like 1 (IL1RL1). The IL1RL1 mediates the biological effects of IL-33, while the IL-18RAcP is required for the high binding affinity of the interleukin 18, which participates in the IFN-gamma production of Th1 cells. Both receptors were shown to be crucial for specifically-induced inflammation, pathogenesis of particular autoimmune disorders and several other organism’s immune responses; therefore, they are thought to be promising therapeutic targets [42]–[44]. Apart from immune-related proteins another essential target molecule was proposed - histamine H2 receptor (HRH2). The HRH2 polypeptide stimulates gastric acid secretion and regulates intestinal secretion, as well as the gastrointestinal motility [45]. Currently, several known therapies use histamine H2 receptor antagonist to cure peptic ulcers (even relapses) and affect gastric acid secretion [46]. Treatments based on the HRH2 antagonist are also commonly used in gastro-esophageal reflux diseases in infants and children [47], [48]. Among the putative plant miRNAs targets, one protein is also important for the therapeutics - nectin-4, alternatively referred to as poliovirus receptor-related protein 4 (PVRL4). This member of the immunoglobulin family have gathered special attention after novel studies showing that the Measles virus, which contributes to over 120,000 child deaths each year, uses nectin-4 protein as a receptor to infect and spread through airway epithelial cells [49]–[51]. The complete detailed information about the aforementioned, interesting molecules and potential biological impact of their gene expression inhibition by particular plant miRNAs can be found in Table 2. To ensure that proposed targets are important and to determine whether they participate in the same biological processes, the predicted molecules were mapped on metabolic pathways from the KEGG database. This part of the analysis showed that several putative targets may be involved in starch, purine, sphingolipid, drug and amino acid metabolism or in more general processes, e.g., transport, immune responses and transcription regulation. Thus, the identified plant-derived miRNAs may represent novel molecular modulators of the aforementioned human biological pathways.

**Table 2:** List of Several Interesting Putative Human Targets for Select Plant miRNAs and Potential Impact of These Food-Derived Molecules on Human Organism.

Protein Name	TargetGeneSymbol	PlantmiRNA	TargetFunction	Potential BiologicalImpact of TargetGene ExpressionInhibition	Ref.
Zing fingerE-box-bindinghomeobox 1	ZEB1	ath-miR319b	Repression ofT-lymphocyte specificIL-2 gene expression	Induction of themesenchymal-to-epithelial transition incancer cell lines; Reductionof cancer cell linesaggressiveness	[40], [41]
Interleukin-1receptor-like 1	IL1RL1	ath-miR166a	Receptor of IL-33	Inhibition of the Th2-typeimmune responses;Reduction of the non-Th2-typeinflammations(e.g. allergic diseases)	[42], [43]
Interleukin-18 receptoraccessory protein	IL18RAP	ath-miR319b	Essential for IL-18binding to its receptorcomplex; IL-18-dependentactivation of NF-kappa-Band JNK	Inhibition of theTh1-mediated inflammatorypathologies	[44], [74]
Histamine H2 receptor	HRH2	zma-miR156a	Mediation of gastric acidsecretion; Regulation ofgastrointestinal motilityand intestinal secretion	Reduction of gastric acidsecretion; Reduction ofulcer incidence; Blockingthe histamine effects	[45]–[47], [75]
Poliovirus receptor-related protein 4(nectin-4)	PVRL4	ctr-miR167	Involvement in celladhesion through thetrans-homophilic and-heterophilic interactions	Blocking the Measlesvirus infection	[49]–[51]

## Conclusions

Our study shows that plant miRNA molecules are abundant in human and porcine breast milk exosomes. The analysis herein using publicly available, high-throughput sequencing data revealed that the food-derived small RNA composition primarily includes conserved plant miRNAs species and is similar to the composition identified by Zhang *et al.* in human serum. What is more, the aforementioned molecules may regulate potential human targets genes important in several biological pathways. Although additional experimental evidence is necessary, our analysis may shed new light on exogenous-origin miRNAs, their stability under harsh conditions and potential roles in living organisms. Moreover, our data support a closer look at plant-derived molecules and their properties. Conclusively, the analysis herein shows that the miRNA “world” is broader than previously thought and suggests a novel field that awaits exploration.

## Materials and Methods

### Data Collection

The 12 raw, small RNA sequencing datasets from porcine and human breast milk exosomes studies were collected from the NCBI Gene Expression Omnibus database records (GEO, http://www.ncbi.nlm.nih.gov/geo/), available under the accession numbers GSE36590 and GSE32253, respectively. The aforementioned datasets comprised 8 small RNA subsets from milk exosomes of 3 female pigs (from six lactigenous stages, 0, 3, 7, 14, 21 and 28 days after birth) and 4 small RNA subsets from milk exosomes of 4 healthy women (60 days after birth). Each of small RNA subset was generated by single-end sequencing in 36 bp reads using the Illumina Genome Analyzer II according to manufacturer’s instructions [19], [20]. The sample preparation protocol was similar in both studies [19], [20] and included methods, and solutions commonly used in similar experiments [28]–[32]. Briefly, the protocol comprised:

- breast milk samples collection and storing at −80°C until analyzed,
- centrifugations and further filtering to eliminate fat globules, cells and cellular debris,
- exosomes isolation by the ExoQuick precipitation procedure (System Biosciences Inc., USA),
- total RNA extraction using the TRIzol-LS (Invitrogen, USA) according to manufacturer’s instructions,
- small RNAs analysis with the Agilent Bioanalyzer 2100 and the RNA 6000 Nano LabChip Kit (Agilent, USA),
- small RNAs fraction isolation by the polyacrylamide gel electrophoresis (PAGE),
- Illumina adaptors ligation and conversion to cDNA by the RT-PCR.

### Bioinformatics Analysis of Small RNA Tags

Each sRNAs dataset was individually, bioinformatically analyzed to clean, remove unnecessary tags and identify plant miRNAs sequences. The full workflow for this analysis is shown in Figure 1.

In the first step of the raw data processing, the adaptor sequences were removed from each read. Then, all low-quality tags were eliminated from the datasets, exactly the sequences with: any N bases, more than 4 bases whose quality score was lower than 10 and more than 6 bases whose quality score was lower than 13. The reads shorter than 17 nucleotides, with a poly A tail, with 5′ primer contaminants and missing insert tags or a 3′ primer were also excluded. The remaining reads were combined into one kind and counted. Next, the sequences that show significant similarity to plant miRNAs were selected. The plant miRNA sequences were downloaded from the Plant MicroRNA Database (PMRD, release of June 2012, http://bioinformatics.cau.edu.cn/PMRD/) [52] and the BlastN method was used to find tags without any gaps and mismatches in the alignment, and with sequence coverage that differed by no more than one nucleotide. The E-value threshold was set at 0.01. The reads selected at this step were assumed as plant miRNAs sequences and two additional verification stages of the analysis were performed to confirm their exogenous origin. First, each annotated sequence that was highly likely a human or pig small non-coding RNA was filtered from the potential plant miRNAs reads. Herein, the *Homo sapiens* and *Sus scrofa* tRNAs, rRNAs, snoRNAs, and snRNAs (available at the Rfam 11.0 release and ENSEMBL 71.0 release) as well as pre-miRNAs and miRNAs sequences (obtained from the miRBase 20.0 release) [53] were collected. The similarity between the potential plants-origin reads and the aforementioned ncRNAs was investigated, individually for each specie, using the BlastN method with the E-value threshold of 0.01; gaps and mismatches were allowed. The tags homologous to the pig and human ncRNAs, respectively, were discarded from the study. A similar search was performed to eliminate repeat-associated sequences and exon fragments. The *H. sapiens* and *S. scrofa* mRNAs and repeat-associated RNAs were downloaded from the NCBI database (April 2013, http://www.ncbi.nlm.nih.gov), and Repbase (17.11 release), respectively. Additionally, the human coding sequences (CDS) were obtained from the NCBI CCDS Database (release 11.0, http://www.ncbi.nlm.nih.gov/CCDS/CcdsBrowse.cgi) [54]. After this procedure, the remaining tags were verified in a second step, wherein the reads were mapped to the human and pig genomes, respectively. The Bowtie 0.12.8 software (http://bowtie-bio.sourceforge.net) [55] with one mismatch allowed were used to map the reads (the potential plant miRNAs) to the *S. scrofa* (Sscrofa9.53, ftp://ftp.sanger.ac.uk/pub/S_scrofa/assemblies/Ensembl_Sscrofa9/) and *H. sapiens* genomes (hg19), respectively. By removing tags that perfectly or near perfectly mapped to the aforementioned genomes, the sets of putative plant miRNA species have been reduced. Considering the RNA editing process, which has been observed in mammals [56]–[58], the potential plant miRNAs sequences were once again mapped to the relevant genomes; however, in this step two mismatches were allowed including one that represent common AI edits (e.g. A>G and T>C). If the sequence has been successfully mapped to the reference genome, it was eliminated from the plant miRNAs dataset. To exclude the possibility that selected small RNAs sequences originated from bacteria, fungi or Archaea, which have been reported as abundant in human plasma and the gastrointestinal tract [22], [59], the remaining reads were compared to sequences from human blood, oral, GI tract and skin microbiomes. The reference genomes were downloaded from the Human Microbiome Project website (HMP, http://www.hmpdacc.org/HMRGD/) [60] and further aligned to the tags using the BlastN method. The E-value threshold was set at 0.01; gaps and mismatches were not allowed. All reads that met the given criteria were discarded from the study. Finally, the remaining tags, which were observed no less than 5 times, were considered as credible plant miRNA sequences. To verify the reliability of the analyzed data and highlight certain variations in plant miRNAs profiles across healthy individuals, the Pearson’s correlations were calculated for human and pig samples, respectively.

### Potential Human Targets Predictions for the Plant miRNAs

Since recent report on particular plant miRNA indicates its function as mammalian LDLRAP1 protein expression level regulator [21], it was interesting to designate human mRNAs that may probably interact with the select identified food-derived miRNAs, potentially transferred from milk to infant’s body *via* the GI tract. For this reason, the putative *H. sapiens* targets predictions were performed using three different computational methods: miRanda (http://www.microrna.org/microrna/getDownloads.do) [61], RNAhybrid 2.1 (http://bibiserv.techfak.uni-bielefeld.de/rnahybrid/dl_pre-page.html) [62] and PITA software (http://genie.weizmann.ac.il/pubs/mir07/mir07_exe.html) [63]. The miRanda search procedure examines sequence complementarity, interspecies conservation and thermodynamic stability of the miRNA:mRNA duplex, while RNAhybrid 2.1 is a tool for finding the minimum free energy (MFE) of target and short RNA (e.g., miRNA) hybridization. The PITA algorithm is based on the miRNA:target interaction model, which calculates the difference between free energy of the miRNA:mRNA bound and the unbound (ΔΔG) state. The described methods were successfully used in many human miRNAs studies [64]–[69] and together, they cover most of the known characteristics of miRNA:target interaction, namely the: seed complementary, interspecies conservation, free energy, target-site accessibility and target-site abundance [70]. For each program, specific rules and restrictions were set up. The prediction parameters of the miRanda method were as follows: (1) G:U base pairing was permitted but scored lower (score +2) than canonical base pairs (score +5), (2) the alignments with gaps and non-canonical base pairs in the “seed” regions (2–8 nt at the 5′ end of the molecule) were discarded, and (3) alignments with scores over 130 and MFE of the structure less than −17 kcal/mol were selected. The RNAhybrid 2.1 selected human mRNAs where the following applied: (1) the hybridization MFE was equal to or below −17 kcal/mol, (2) the maximum bulge loop size was 2 nucleotides, and (3) the maximum internal loop size was 2 nucleotides. The PITA targets search considered only sequences where the 7-8-mer “seed” region did not include mismatches and G:U base pairs, while their calculated ΔΔG scores were below −10. The *H. sapiens* 3′ UTR, 5′ UTR and CDS sequences, that served as potential plant miRNAs targets, were downloaded from the UCSC Bioinformatics Site (April 2013, http://genome.ucsc.edu/index.html) and NCBI CCDS Database, respectively. Using the intersection of all three methods a consensus list of putative human targets was generated and further sorted by the highest alignment score and lowest MFE of the structure. The top best 20–25 hits were collected. To designate potential processes involving the predicted mRNA sequences and to suggest a probable influence of food-derived plant miRNAs on human organism, the selected targets were annotated using the Blast2GO (http://www.blast2go.com/b2ghome) [71] and DAVID v6.7 tools (http://david.abcc.ncifcrf.gov/home.jsp) [72]. In the analysis by Blast2GO software, the GO terms were obtained based on the BlastX search against the “nr” NCBI database with the E-value threshold of 1e^−6^. The KEGG (Kyoto Encyclopedia of Genes and Genomes) [73] database was also searched; the E-value threshold was sat at 1e^−10^. The best hits from each annotation were collected.

## Supporting Information

File S1 Contains the following files: **Table S1.** The plant miRNAs identified in sample 1 of porcine breast milk exosomes collected 0 days after birth. **Table S2.** The plant miRNAs identified in sample 2 of porcine breast milk exosomes collected 0 days after birth. **Table S3.** The plant miRNAs identified in sample 3 of porcine breast milk exosomes collected 0 days after birth. **Table S4.** The plant miRNAs identified in sample of porcine breast milk exosomes collected 14 days after birth. **Table S5.** The plant miRNAs identified in sample of porcine breast milk exosomes collected 21 days after birth. **Table S6.** The plant miRNAs identified in sample of porcine breast milk exosomes collected 28 days after birth. **Table S7.** The summary list of plant-derived miRNAs identified in six samples of porcine breast milk exosomes (collected 0, 14, 21 and 28 days after birth) together with their calculated average reads counts. **Table S8.** Plant MIR families identified in six samples of porcine breast milk exosomes collected 0, 14, 21 and 28 days after birth.
(XLS)

File S2 Contains the following files: **Table S9.** The plant miRNAs identified in sample 1 of human breast milk exosomes. **Table S10.** The plant miRNAs identified in sample 2 of human breast milk exosomes. **Table S11.** The summary list of plant miRNAs identified in two samples of human breast milk exosomes together with their calculated average reads counts. **Table S12.** Plant MIR families identified in two samples of human breast milk exosomes.
(XLS)

File S3 Contains the following files: **Table S13.** List of the *H. sapiens* best potential targets predicted for five select plant miRNAs. **Table S14.** List of the potential KEGG processing pathways, in which the five select plant miRNAs may participate in human organism.
(XLS)
